# Long Noncoding RNA-LET Suppresses Tumor Growth and EMT in Lung Adenocarcinoma

**DOI:** 10.1155/2016/4693471

**Published:** 2016-11-08

**Authors:** Bin Liu, Chun-Feng Pan, Zhi-Cheng He, Jun Wang, Peng-Li Wang, Teng Ma, Yang Xia, Yi-Jiang Chen

**Affiliations:** Department of Thoracic and Cardiovascular Surgery, The First Affiliated Hospital of Nanjing Medical University, Nanjing, Jiangsu 210029, China

## Abstract

Recently, many studies showed that long noncoding RNAs (lncRNAs) are involved in tumor progression. It is reported that lncRNA-LET is downregulated and has antitumor effect on several types of cancer. This study focuses on the role of lncRNA-LET on lung adenocarcinoma (LAC) progression. RT-PCR results indicated that frequent downregulation of lncRNA-LET in LAC tissues was related to clinicopathologic factors. lncRNA-LET knockdown significantly promoted LAC cell proliferation, invasion, and migration while lncRNA-LET overexpression obviously inhibited LAC cell proliferation, invasion, and migration, indicating a tumor inhibition of lncRNA-LET in LAC progression. Besides, lncRNA-LET inhibited EMT and negatively regulated Wnt/*β*-catenin pathway in part. Our study suggests that lncRNA-LET exhibits an important tumor-suppressive effect on LAC progression by inhibiting EMT and Wnt/*β*-catenin pathway, which provides potential therapeutic targets for LAC.

## 1. Introduction

As the leading cause of cancer death, there were about 1,800,000 new lung cancer cases' occurrence in 2012, about 13% of total cancer diagnoses [1]. Lung cancer mainly contains two types: small cell lung cancer (SCLC) and non-small-cell lung cancer (NSCLC), accounting for 15% and 85% of total lung cancer diagnoses, respectively [2]. As the most widespread histological type of NSCLC, lung adenocarcinoma (LAC) leads to more than 500,000 deaths every year around the world [3]. Therefore it is of great clinical value to further reveal the molecular mechanisms of LAC progression and development and develop new therapeutic targets for LAC patients.

As a type of long noncoding RNA transcripts, long noncoding RNAs (lncRNAs) are longer than 200 nucleotides in length [4]. Increasing evidences have shown that lncRNAs are involved in tumor progression [5–7].

LncRNA-Low Expression in Tumor (lncRNA-LET), a recently identified lncRNA located at chromosome 15q24.1, has been demonstrated to be downregulated and plays a tumor-suppressive role in several types of cancer, such as hepatocellular carcinomas, nasopharyngeal carcinoma, cervical cancer, and gallbladder cancer [8–11]. However, to our knowledge, the effect of lncRNA-LET in progression and development of LAC is still unknown.

In this study, we detected the expression level of lncRNA-LET in LAC tumor tissues and paracarcinoma tissues and investigated its relationship with clinicopathologic factors in LAC. Then, tumor inhibition of lncRNA-LET was explored* in vitro* and* in vivo*. Moreover, we also assayed the molecular marker levels of EMT and *β*-catenin to reveal inhibition of lncRNA-LET on EMT and Wnt/*β*-catenin pathway. Therefore, this study showed that lncRNA-LET has a significant role in LAC progression and development and may act as a potential therapeutic target for LAC.

## 2. Materials and Methods

### 2.1. Patients and Tissue Samples

In all, 60 LAC tissues and matched paracarcinoma tissues used in this study were obtained from patients undergoing thoracoscopic and open cardiac surgery in Jiangsu Province People Hospital from 2013 to 2014. In this study, LAC patients had not received chemotherapy or radiotherapy treatment. All surgical specimens were immediately frozen in liquid nitrogen until use. This research was approved by Jiangsu Province People Hospital and had received informed consents from all patients. LAC patients were staged in accordance with the tumor node metastasis (TNM) staging system (the 7th edition) of the AJCC staging system.

### 2.2. Cell Culture

16HBE cells, PC-9 cells, H358 cells, SPCA1 cells, H1975 cells, H1299 cells, and A549 cells were cultured in RPMI1640 medium containing 10% fetal calf serum, 100 U/mL penicillin, and 100 *μ*g/mL streptomycin, within a humidified atmosphere at 37°C containing 5% CO_2_.

### 2.3. Plasmid and Transfection

For overexpression of lncRNA-LET in H1299 cells, the lncRNA-LET gene was cloned into the lentiviral-vector pCDH-CMV-MCS-EF1-copGFP (System Biosciences, Mountain View, CA) and the primer sequences are as follows: lncRNA-LET: sense, 5′-GTTGTTGTTGCATTGGGGT-3′, antisense, 5′-AAGATGGAGAGTGGAGCCT-3′. H1299 cells were transfected with LV-Vector or LV-LncRNA-LET. For knockdown of lncRNA-LET in A549 cells, the sequences of shRNA targeting for lncRNA-LET were designed as 5′-GGAGGGUGCUUGACAAUAAUU-3′ and the NC-shRNA was purchased from Genechem (Shanghai, China). A549 cells were transfected with NC-shRNA or LncRNA-shRNA.

### 2.4. RNA Extraction and qRT-PCR

lncRNA-LET and nuclear *β*-catenin expression level were measured by qRT-PCR. In brief, trizol reagent (Invitrogen, Carlsbad, CA) was used to extract the RNA according to the manufacturer's instruction. Reverse Transcription Kit (Takara, Dalian, China) was used to synthesize cDNAs. The real-time reverse transcription PCR was carried out by using Power SYBR Green (Takara, Dalian, China). GAPDH was used as an internal control. qRT-PCR and data collection were carried out by the ABI7500 system (Applied Biosystems, Foster, CA). The relative levels of mRNAs were calculated using the 2^−ΔΔCt^ method: lncRNA-LET: 5′-CCTTCCTGACAGCCAGTGTG-3′ (forward), 5′-CAGAATGGAAATACTGGAGCAAG-3′ (reverse); *β*-catenin: 5′-GCTTTCAGTTGAGCTGACCA-3′ (forward), 5′-CAAGCTCAAGATCAGCAGTCTC-3′ (reverse); GADPH: 5′-GTCAACGGATTTGGTCTGTATT-3′ (forward), 5′-AGTCTTCTGGGTGGCAGTGAT-3′ (reverse).

### 2.5. Cell Proliferation

Cells were seeded onto 96-well plate and incubated overnight at 37°C containing 5% CO_2_. 20 *μ*L of MTT solution per well was added into the 96-well plate and cultured for 4 h at 37°C. Then 150 *μ*L DMSO was added into the 96-well plate and incubated for 10 min. The absorbance was read by an enzyme-linked immunosorbent assay (ELISA) reader at 490 nm.

### 2.6. Wound Healing Assay

Cells were spread at the bottom of 12-well plates marked by a horizontal line on the back for targeting the same field of vision; a line wound was made by scraping 100 *μ*L tips across the confluent cell layer. After sucking cell culture and washing the cells for three times to remove detached cells and debris, the serum-free medium was added into the 12-well plates and incubated under the usual culture conditions for 48 h; wound closure was captured using a light microscope (DFC500; Leica, Wetzlar, Germany). AxioVision version 4.7 software (Carl Zeiss Meditec, Dublin, CA, USA) was used to measure the wound closure.

### 2.7. Transwell Assay

Cell invasion assay was operated by polycarbonate membrane Boyden chamber insert (EMD Millipore, Billerica, MA, USA). The transfected cells were added into the upper invasion chamber; the 500 *μ*L culture medium was added into the lower invasion chamber. 45 *μ*g Matrigel (BD Biosciences; San Jose, CA, USA) was used to precoat each insert. The Matrigel invasion chambers were incubated under the usual culture conditions for 48 h. After removing the filter inserts and the cells on the upper side of the filter, the invaded cells on the lower chamber were suspended in 100% precooling methanol for 10 min. Then cells were stained with 0.05% crystal violet for 30 min, washed with PBS buffer, and captured using microscopic inspection (DFC500; Leica) according to the manufacturer's instructions. Finally, the values for invasion were carried out under three fields per membrane.

For cell migration assay by transwell assay, each insert needed was not precoated with Matrigel. The other steps were similar to cell invasion assay.

### 2.8. Western Blot

In order to investigate expression level of *β*-catenin, E-cadherin, N-cadherin, c-Myc, and COX-2, cells were collected and cell lysis was performed by using RIPA lysis buffer (Cell Signal Technology, Danvers, MA) including protease inhibitors on ice according to the manufacturer's instruction. The extracted protein was quantified by bicinchoninic acid quantification assay (Pierce Biotechnology, Inc., Rockford, IL, USA). Then, the total cellular proteins were subjected to SDS-PAGE (10%) for western analysis. After transferring to polyvinylidene difluoride (PVDF) membranes, blots were incubated with 5% BSA in Trisbuffered saline containing 0.5% Tween 20 for 60 min and incubated overnight at 4°C on a rocker with the following primary antibodies: polyclonal rabbit anti-human *β*-catenin antibody (1 : 1,000), monoclonal mouse anti-human E-cadherin antibody (1 : 1,000), polyclonal rabbit anti-human N-cadherin antibody (1 : 1,000), monoclonal rabbit anti-human c-Myc antibody (1 : 1,000), and polyclonal rabbit anti-human COX-2 antibody (1 : 1,000). Following washing three times with TBS-T for 5 min, the blots were incubated with horseradish peroxidase- (HRP-) conjugated goat anti-rabbit IgG H&L polyclonal secondary antibody (1 : 1,000) at room temperature for 1 h. All antibodies were purchased from Abcam (Cambridge, MA, USA). The bands were captured under an enhanced chemiluminescence detection system (GE Healthcare Life Sciences).

### 2.9. Xenograft Model

All mice experiments were operated with the approval of the Animal Studies Ethics Committee of the First Affiliated Hospital of Nanjing Medical University according to the Guide for the Care and Use of Laboratory Animals published by the US National Institutes of Health (NIH publication number 85-23, revised 1996). H1299 cells (6 × 10^4^/mL) transfected with LV-LncRNA-LET or LV-Vector were implanted into the flanks of NOD/SCID mice (4-5 weeks old) subcutaneously. The tumor volumes were evaluated by using calipers. The mice were sacrificed and the grafts were removed after 6 weeks.

### 2.10. HE Staining

The grafts were fixed by formalin and embedded by paraffin. Tumor tissue sections were cut to 4 *μ*m thickness and mounted in Poly-Lysine (Sigma, USA) coated glass slides. Sections were deparaffinized in xylene, dehydrated in a decreasing ethanol series, and performed by HE staining.

### 2.11. Immunohistochemistry

After sections were deparaffinized and dehydrated, sections were immersed in methanol with 0.3% (vol/vol) H_2_O_2_ for 30 min. Sections were heated in citrate buffer (10 mM, pH 6.0) at 120°C for 5 min. Then the sections were incubated with primary antibody (polyclonal rabbit anti-human PCNA antibody) overnight at 4°C. After washing with PBS, sections were incubated with biotinylated secondary antibody at room temperature for 1 h. Diaminobenzidine (0.05% for 10 min at temperature) was employed to be as chromogen. Slides were stained with Mayer's hematoxylin solution and mounted in Entellan (Merck KGaA, Darmstadt, Germany).

### 2.12. Statistical Analysis

All statistical analyses were carried out by SPSS 17.0 and the data were expressed as the means ± SD. Comparison of lncRNA-LET level between tumors and paracarcinoma was performed using the Wilcoxon test. The correlation between lncRNA-LET level and clinical characteristics was analyzed using the chi-squared test. *P* < 0.05 was considered to indicate a statistically significant difference.

## 3. Results

### 3.1. Expression of lncRNA-LET in LAC Patients and Cell Lines

**Figure 1:**
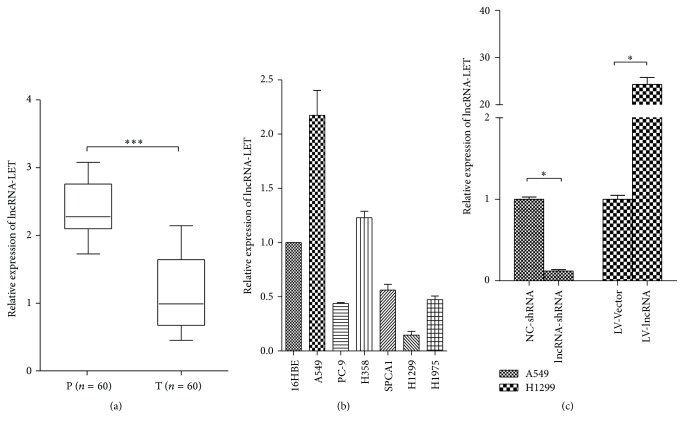
Expression of lncRNA-LET in LAC patients and cell lines. (a) Analysis of lncRNA-LET expression level in paracarcinoma tissue (P) and tumor tissue (T). Total RNA was detected by quantitative real-time PCR (qRT-PCR) and GAPDH was used as an internal control. lncRNA-LET was significantly downregulated in LAC tumor tissues compared with the paracarcinoma tissue. (b) Analysis of lncRNA-LET expression level in seven cell lines was detected by using RT-PCR. GAPDH was used as an internal control. (c) Analysis of transfection efficiency in A549 cells and H1299 cells. lncRNA-LET expression in A549 cell line after being transfected with lncRNA-LET-shRNA or NC-shRNA was determined by qRT-PCR. lncRNA-LET expression in H1299 cell line after being transfected with LV-LncRNA-LET or LV-Vector was determined by qRT-PCR. Data are presented as the mean ± SD of three independent experiments. ^*∗∗∗*^ *P* < 0.001;  ^*∗*^ *P* < 0.05.

The expression of lncRNA-LET in LAC tumor tissues and paracarcinoma tissues from 60 patients was detected by quantitative real-time PCR (qRT-PCR).  Figure 1(a) showed that expression of lncRNA-LET was significantly downregulated in lung adenocarcinoma tissues compared with paracarcinoma tissues (*P* < 0.001), suggesting that frequent downregulation of lncRNA-LET in LAC may be related to LAC pathogenesis. To identify the correlation of lncRNA expression with clinicopathologic factors, we divided the 60 LAC patients into a high level and a low level group according to the mean level of lncRNA-LET. The clinicopathologic factors were analyzed in Table 1. Compared with high level group of lncRNA-LET, low level group of lncRNA-LET was significantly associated with a less differentiated histology, higher tumor stage, and more lymph node metastasis (*P* < 0.05) but not correlated with gender, age, site of tumor, and tumor size (*P* > 0.05).

In addition, we detected the expression level of lncRNA-LET in several normal and LAC cell lines via using RT-PCR and showed that, comparing with 16HBE cell line, A549 and H358 cells expressed the higher level of lncRNA-LET while other cell lines expressed lower level of lncRNA-LET (Figure 1(b)). To explore the role of lncRNA-LET in LAC progression and development, we chose A549 cell line for lncRNA-LET knockdown and H1299 cell line for lncRNA-LET overexpression, and the transfection efficiency was subsequently detected via RT-PCR as shown in Figure 1(c). The lncRNA-LET expression was effectively suppressed in A549 cells by lncRNA-shRNA and elevated in H1299 cells by LV-lncRNA.

### 3.2. Effects of lncRNA-LET on LAC Cell Proliferation, Migration, and Invasion

**Figure 2:**
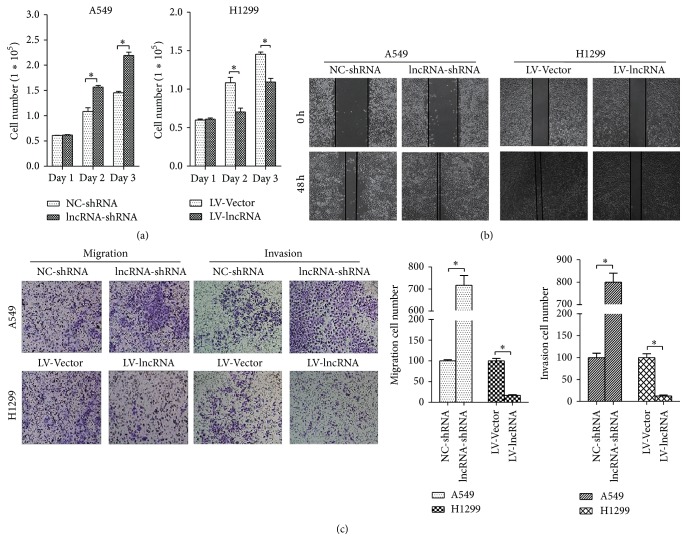
Effects of lncRNA-LET on LAC cell proliferation, migration, and invasion. (a) MTT assays were performed to detect the proliferation of A549 cells and H1299 cells. (b) Wound healing assays were performed to determine the migration of A549 cells and H1299 cells. (c) Transwell assay was employed to assay the migration and invasion of A549 cells and H1299 cells. A549 cells were transfected with lncRNA-LET-shRNA or NC-shRNA and H1299 cells were transfected with lncRNA-LET-shRNA or NC-shRNA. Data are presented as the mean ± SD of three independent experiments. ^*∗*^ *P* < 0.05.

Since lncRNA-LET is downregulated in LAC and associated with the progression of LAC, we next explored the role of lncRNA-LET in LAC cell lines. As showed in Figure 2(a), MTT assays showed that lncRNA-LET knockdown significantly promoted cell proliferation of A549 cells while lncRNA-LET overexpression obviously inhibited cell proliferation of H1299 cells. Then, wound healing assay was employed to determine cell migration. Compared with the control group, A549 cells with lncRNA-LET knockdown exhibited stronger migration while overexpression of lncRNA-LET significantly impaired migration ability in H1299 cells (Figure 2(b)). On the other hand, as showed in Figure 2(c), effects of lncRNA-LET on cell migration by using transwell assay were the same as that performed by wound healing assay. Besides, Transwell assay also revealed effects of lncRNA-LET on cell invasion (Figure 2(c)), showing that lncRNA-LET knockdown markedly accelerated A549 cell invasion and lncRNA-LET overexpression obviously inhibited H1299 cell invasion. These results demonstrated that lncRNA-LET might be involved in progression and development of LAC.

### 3.3. lncRNA-LET Inhibited EMT and the Canonical Wnt/*β*-Catenin Pathway

**Figure 3:**
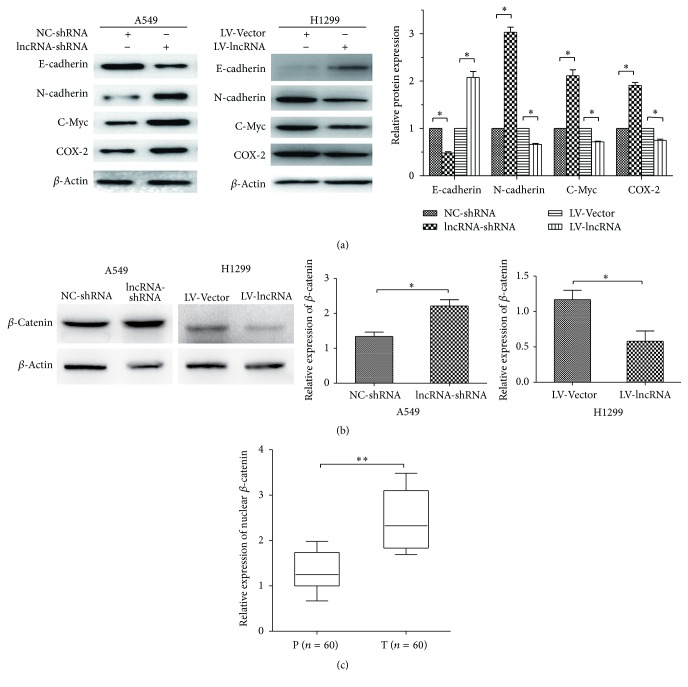
lncRNA-LET expression inhibited EMT and canonical Wnt/*β*-catenin pathway in part. (a) Western blot was performed to assay the molecular marker levels of EMT. (b) Western blot was performed to assay the protein level of nuclear *β*-catenin in LAC cell lines. The relative protein levels were normalized to *β*-actin. A549 cells were transfected with lncRNA-LET-shRNA or NC-shRNA and H1299 cells were transfected with lncRNA-LET-shRNA or NC-shRNA. Relative protein expression was quantified by the Image J software. (c) RT-PCR was performed to assay the mRNA level of nuclear *β*-catenin in paracarcinoma tissue (P) and tumor tissue (T). Data are presented as the mean ± SD of three independent experiments. ^*∗∗*^ *P* < 0.01; ^*∗*^ *P* < 0.05.

To demonstrate whether the lncRNA-LET affected EMT, we employed western blot to assay the molecular marker levels of EMT.  Figure 3(a) displayed that lncRNA-LET knockdown inhibited E-cadherin level and elevated levels of N-cadherin, c-Myc, and COX-2 in A549 cells while lncRNA-LET overexpression elevated levels of E-cadherin and inhibited levels of N-cadherin, c-Myc, and COX-2 in H1299 cells, indicating that lncRNA-LET could inhibit EMT.

The Wnt/*β*-catenin signaling pathway plays an important role in the development of EMT and cancer metastasis [12]. Thus we further determine the *β*-catenin protein level in cell nucleus by using western blot. We found that A549 cells with lncRNA-LET knockdown exhibited more accumulation of *β*-catenin than control group, and H1299 cells with lncRNA-LET overexpression showed a contrary tendency (Figure 3(b)). Besides, the mRNA expression of nuclear *β*-catenin in LAC tissues was also detected by using RT-PCR and we demonstrated that accumulation of nuclear *β*-catenin in LAC tissues compared with paracarcinoma tissues (Figure 3(c)). Taken together, these results suggested that lncRNA-LET expression inhibited EMT and the canonical Wnt/*β*-catenin pathway in part.

### 3.4. Effects of lncRNA-LET on LAC Tumor Growth* In Vivo*

**Figure 4:**
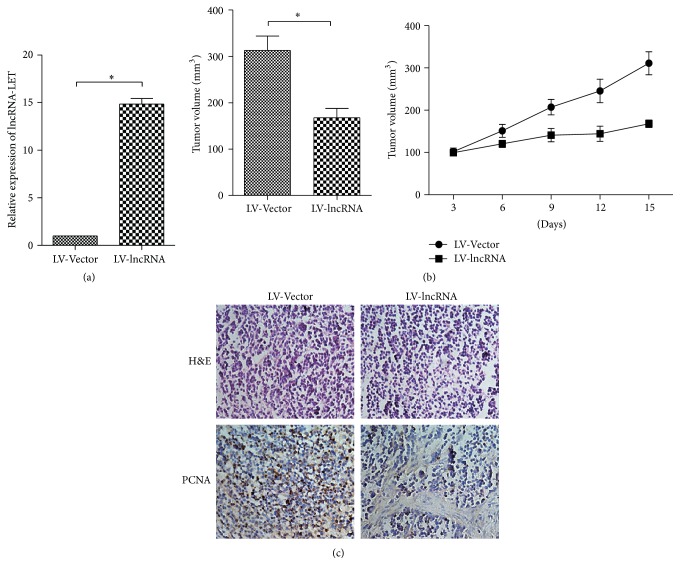
lncRNA-LET inhibits LAC tumor growth and cell proliferation* in vivo*. (a) Analysis of lncRNA-LET expression level in tumor tissues was detected by using RT-PCR. GAPDH was used as an internal control. (b) Tumor volume in lncRNA-LET overexpression group was determined by using calipers. (c) HE and immunohistochemical staining showed that lncRNA-LET overexpression inhibited the aggressive phenotype of LAC cells* in vivo*, as indicated by the expression of PCNA-positive cells. Data are presented as the mean ± SD of three independent experiments. ^*∗*^ *P* < 0.05.

H1299 cells transfected with LV-lncRNA-LET or LV-Vector were injected into mice subcutaneously. lncRNA-LET expression in tumor tissues was detected by qRT-PCR and showed the lncRNA-LET overexpression in LV-lncRNA-LET group compared with that in LV-Vector group (Figure 4(a)). Tumor volume in lncRNA-LET overexpression group was significantly lower than tumor in the control group (Figure 4(b)). Immunohistochemical staining of tumor tissues showed that there was a decrease in proliferation marker (PCNA) in LV-lncRNA-LET group versus LV-Vector group (Figure 4(c)). These data verified the tumor inhibition of lncRNA-LET* in vivo*.

## 4. Discussion

In the present study, we demonstrated that the expression level of lncRNA-LET was significantly downregulated in lung adenocarcinoma tissues compared with adjacent nontumor tissues, and low level group of lncRNA-LET was significantly associated with a less differentiated histology, higher tumor stage, and more lymph node metastasis compared with high level group of lncRNA-LET, suggesting a crucial role of lncRNA-LET in progression and development of LAC. Furthermore, we also found that lncRNA-LET knockdown significantly promoted A549 cell proliferation, invasion, and migration while lncRNA-LET overexpression obviously inhibited H1299 cell proliferation, invasion, and migration. In addition, we further tested the effect of lncRNA-LET in xenograft model and found that lncRNA-LET could inhibit tumor growth and proliferation. Thus, our results demonstrated that lncRNA-LET acts as a tumor suppressor in progression and development of LAC.

Previous studies have showed that EMT was involved in cell motility and invasiveness in several types of cancer progression, including LAC, and control or suppression of tumor cell epithelial-mesenchymal transition (EMT) could significantly inhibit metastasis and growth of LAC [13–16]. EMT is a conserved cellular process in which epithelial tumor cell lacks its polarity and transforms into a mesenchymal phenotype [16, 17]. The feature of EMT occurrence is that the epithelial marker E-cadherin is downregulated and mesenchymal marker, like N-cadherin, is upregulated [18, 19]. To demonstrate whether the lncRNA-LET affected EMT, we detected the molecular marker levels of EMT and showed the inhibition of lncRNA-LET on EMT.

Recently, it has been reported that overexpression of Wnt signaling pathway is involved in metastasis of lung cancer [12, 20]. Wnt/*β*-catenin pathway can control cell proliferation by regulating the expression of cell cycle-related gene including c-Myc and modulate EMT by downregulating E-cadherin and upregulating N-cadherin [21, 22]. Besides, accumulation of *β*-catenin in cell nucleus indicates that canonical Wnt/*β*-catenin pathway is activated and related to tumor progression and metastasis [23, 24]. Thus, we further determined the *β*-catenin level in cell nucleus and LAC tissues and found that accumulation of nuclear *β*-catenin in LAC tissues compared with paracarcinoma tissues. Moreover, lncRNA-LET expression could block activation of canonical Wnt/*β*-catenin pathway. In our previous study, we have demonstrated that lncRNA-LET functions as a tumor suppressor gene by activating p53 in esophageal squamous cell carcinoma cells [25]. In addition, p53 acts upstream of Wnt to suppress canonical Wnt signaling pathway [26]. Thus we speculate that lncRNA-LET may suppress Wnt/*β*-catenin pathway by regulating p53 in LAC. Although better evidence is necessary, the present study also supports the rationale of an important role for lncRNA-LET that plays tumor-suppressive roles in progression and development of LAC by inhibiting Wnt/*β*-catenin pathway.

In conclusion, our study demonstrated that lncRNA-LET had tumor-suppressive effect on LAC progression and development* in vitro* and* vivo*, and was correlated with clinicopathologic factors. Besides, lncRNA-LET could inhibit EMT and negatively regulated Wnt/*β*-catenin pathway in part. Our findings suggest that lncRNA-LET can act as a potential therapeutic target for LAC.

## Figures and Tables

**Table 1:** lncRNA-LET expression and clinicopathologic factors in lung adenocarcinoma (LAC).

	lncRNA-LET expression			
Characteristics	Case number	Low	High	*P* value
Gender				
Male	23	11	12	0.791
Female	37	19	18	
Age (year)				
<60	38	20	18	0.592
≥60	22	10	12	
Site of tumor				
Left lung	25	14	11	0.757
Right lung	35	21	14	
Histological grade				
Moderately	36	15	21	0.027^*∗*^
Poorly	24	17	7	
Tumor stage				
I/II	36	14	22	0.015^*∗*^
III/IV	24	17	7	
Lymph node metastasis				
Negative	20	6	14	0.028^*∗*^
Positive	40	24	16	
Tumor size				
T1/T2	38	17	21	0.284
T3/T4	22	13	9	

*∗* indicates  *P* < 0.05.
